# Epidemiology of bronchial asthma and asthma control assessment in Henan Province, China

**DOI:** 10.1186/2213-0802-2-5

**Published:** 2014-03-05

**Authors:** Wenping Zhang, Xianliang Chen, Lijun Ma, Jizhen Wu, Limin Zhao, Hongyan Kuang, Taibo Huang, Jianjian Cheng, Luoxian Zhang, Yong Qi, Beibei Sun, Hongyan Niu

**Affiliations:** Department of Respiratory and Critical Care Medicine, People’s Hospital of Henan Province, Zhengzhou University, Zhengzhou, Henan Province China

**Keywords:** Prevalence, Socioeconomic status, Medications for asthma, Asthma control, Asthma guideline

## Abstract

**Background:**

Prevalence of bronchial asthma, asthma treatment assessment, and estimation of the control level among asthma patients in Henan Province, China are reported in this paper.

**Methods:**

We selected 10 among the 109 cities and districts in Henan province using a multistage stratified cluster random sampling method. A total of 500 households from each city and district were chosen. Approximately 20,000 residents from a total of 5,000 households were randomly selected to answer a questionnaire recommended by the China Asthma Alliance. Asthma patients were asked to answer a detailed questionnaire using the symptom-based guidelines to assess the levels of disease control.

**Results:**

The overall prevalence of asthma was 0.73% ± 0.12%. Urban and rural residents had asthma prevalence rates of 1.1% ± 0.23% (88/7,924) and 0.48% ± 0.12% (57/11,792), respectively. Among the asthma patients, only 33.8% (52) received regular medication, 25% (13) used oral glucocorticoids, and 71.1% (37) used oral theophylline. The classified control levels of patients were as follows: 33.1% controlled, 49.7% partially controlled, and 17.2% uncontrolled. A total of 38.5% and 27.5% of regularly and irregularly treated asthma patients reached controlled level, respectively. The two groups significantly differed in asthma control level.

**Conclusion:**

Asthma prevalence is low in Henan Province, China. Urban residents have higher prevalence of asthma than rural residents do. Patients with asthma receive insufficient medication, resulting in suboptimal asthma control. Improvement in diagnosis and treatment of asthma patients is urgently needed.

## Background

Bronchial asthma is a common chronic disease of the respiratory system that affects approximately 300 million people worldwide [1]. Current Global Initiative for Asthma (GINA) guidelines reported that the prevalence of asthma is estimated to be 1% to 18% [2]. Reports have indicated changes in the overall prevalence of asthma [3–5]. However, a firm conclusion on whether the trend is increasing or declining in a particular country for a certain period is not available [6, 7]. As of this writing, few epidemiological data on national asthma prevalence have been reported in China. The Chinese population has relatively low asthma prevalence. Investigation conducted by the National Prevention and Treatment of Children Asthma Group in 2003, which included 430,000 children aged between 0 and 15 years old in 43 cities, reported that the prevalence of childhood asthma in the urban environment is 1.97% [8]. The estimated incidence of asthma was 1.05% in Henan Province in 2000 [9]. Time trends and regional variations in asthma prevalence are difficult to assess in China because of insufficient data [10]. GINA guidelines have been the main reference source for the national asthma guidelines, and the recommended asthma control classification is widely adopted by general practitioners and respiratory specialists in China in the assessment of disease control among asthma patients. We aim to report the prevalence of asthma among residents in Henan Province in 2010 and to evaluate the treatment and disease control among asthma patients. An assessment of the asthma disease, treatment, and control level was also conducted.

Henan province is located at the eastern part of China (31° 23′ N to 36°22′ N, between 110° 21′ E and 116°39′ E). Henan has 19 cities and is a developing province with a population of approximately 104,890,000 in 2012 [11].

## Methods

### Study sample

This cross-sectional study of randomly selected residents in Henan Province was conducted from June 2010 to January 2011. The sampling unit comprised groups of people living together as families or individuals living alone. The sample size was estimated as follows: overall prevalence was *P* = 1.05% (*α* = 0.05) and estimated total sample size was 10,000. The actual completed sample size was 19,878, which included 10,275 (51.7%) males and 9,603 (48.3%) females.

**Figure 1:**
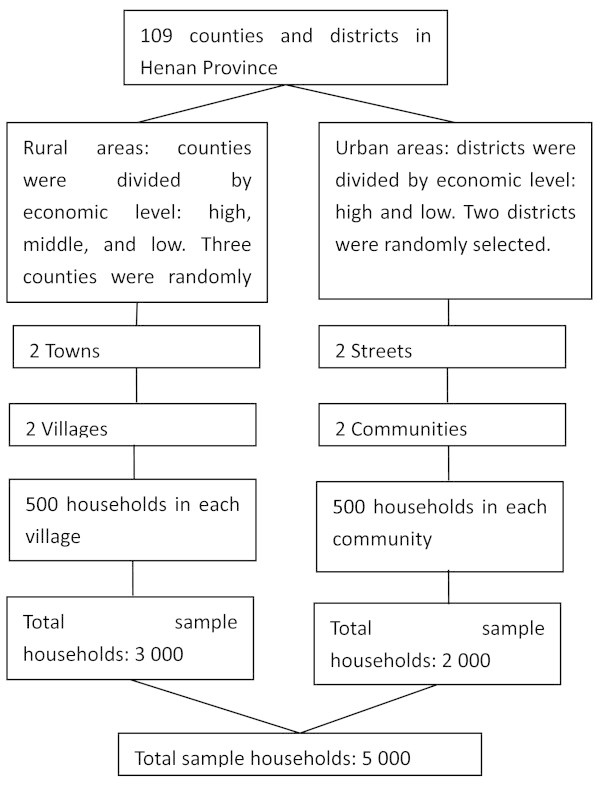
**Sampling flowchart.**

This study used stratified multi-stage cluster random sampling method (Figure 1). Henan Province comprised 109 counties (county level city) and districts. The counties were divided into three groups according to their economic status: high-, middle-, and low-level groups [12]. Districts were divided into two groups according to economic status: high- and low-level groups [12]. Three counties (one county from each group) and two districts (one district from each group) were randomly selected. Two towns were selected in each county, and two streets were selected in each district. Two administrative villages were selected in each township and two communities in each street. Finally, 500 household residents were randomly selected in each administrative village and community. A total of 3,000 households in rural areas and 2,000 households in urban areas were sampled. The sample was weighed to rural areas (2,000 vs. 3,000 subjects) for population in urban areas accounted for 60% of the total in Henan province. Each investigated group comprised field investigators, and survey instructors. Field investigators were trained medical interns and residents. They surveyed each household member using questionnaires. Survey instructors, who were associated chief respiratory physicians and higher-ranking medical practitioners, were responsible for organization, guidance, inspection, and quality control. We adopted the asthma questionnaire recommended by the China Asthma Alliance. The survey included general condition questions, an asthma-screening questionnaire, and a questionnaire for asthma patients. The investigation period was from April 2010 to October 2010.

### Asthma diagnosis

The diagnostic criteria for bronchial asthma [13] used in both questionnaires and lung function tests included the following items. The subject should have been previously diagnosed with asthma. If the subject had no asthma history, the following 10 questions (Qs) were asked: Q1. Have you had recurrent (1) wheezing, (2) coughing, (3) breathlessness, (4) dyspnea, or (5) chest tightness more than three times?; Q2. Do you often have night or morning attacks of the symptoms in Q1?; Q3. Have you ever been awakened at night by these symptoms?; Q4. Have you ever experienced difficulty of breathing or retarded expiratory flow?; Q5. Do you hear wheezing sounds from your chest whenever these symptoms occur?; Q6. Do your symptoms resolve spontaneously?; Q7. Do your symptoms disappear after taking salbutamol or theophylline?; Q8. Do your symptoms occur or worsen during a certain season?; Q9. Do your immediate family members have asthma?; and Q10. Do your symptoms occur during or after exercise? These criteria for asthma are listed in Table 1. For suspected asthma patients, a lung function test was arranged in our hospital to establish a diagnosis. An increase in FEV_1_ of ≥12% and ≥200 ml after administration of a bronchodilator indicates reversible airflow limitation consistent with asthma.

**Table 1:** **Criteria for asthma in this study**

	Asthma		Suspected asthma	
Q1 (1)	Yes	No	Yes	No
Q1 (2)–(5)		Yes		Yes
Q2–Q10	Yes ≥ 5	Yes ≥ 6	Yes ≥ 3, but < 5	Yes ≥ 4, but < 6

### Asthma control

Asthma control was assessed for 4 weeks using the asthma control classification recommended by GINA. Table 2 describes the characteristics of controlled, partially controlled, and uncontrolled asthma.

**Table 2:** **Levels of asthma control**

Assessment of current clinical control (preferably over 4 weeks)			
Characteristics	Controlled (all of the following)	Partially controlled (any measure present)	Uncontrolled
Daytime symptoms	None (twice a week or less)	More than twice a week	Three or more features of partially controlled asthma
Limitation of activities	None	Any	
Nocturnal symptoms	None	Any	
Need for relief/rescue treatment	None (twice a week or less)	More than twice a week	

### Statistical analysis

Prevalence rates with 95% confidence intervals were estimated for all participants in each sample area and for participants in each age group. An *Χ*^2^ test was used for prevalence comparison across the two groups. A *P* value of less than 0.05 was considered statistically significant.

All data analyses were performed using SPSS version 19.0 (SPSS, Inc., Chicago, IL).

## Results

### Prevalence of asthma

Among the 19,861 participants in this study, 51.7% (10,275) were males. Table 3 summarizes the demographic and health profiles of the sampling areas. Sex did not vary significantly, but education level, smoking habit, and medical insurance of the residents varied between the rural and urban sampling areas. A total of 145 people were diagnosed as asthma sufferers, including 79 males. The total morbidity rate was 0.73% ± 0.12%, and prevalence rates of male and female were 0.76% ± 0.17% and 0.69% ± 0.17%, respectively. Table 4 lists the prevalence rates in each sampling area.

**Table 3:** **Sample characteristics in Henan Province from January 2010 to June 2010**

	Urban		Rural			P-value
	Zhongyuan district in Zhengzhou (economic level – H)	Longting District in Kaifeng (economic level – L)	Zhongmu county (economic level – H)	High-tech zone in Puyang City (economic level – L)	Mengjin County (economic level – M)	
Subjects (n)	4,017	3,907	4,180	3,894	3,718	
Females	48.8%	49.5%	48.1%	48.0%	48.8%	0.686
College degree	4.3%	8.8%	0.9%	1.2%	1.2%	〈0.01
Without medical insurance	0.4%	4.5%	1.1%	0.3%	0.5%	〈0.01
Smoke or smoked in the past	16.2%	21.1%	23.1%	22.5%	21.1%	〈0.01

**Table 4:** **Asthma prevalence in each sampling area in Henan Province from January 2010 to June 2010**

Sampling area	No. of samples	Cases of asthma	Prevalence of asthma (%)	***P*** value
Urban area	7,924	88	1.11 ± 0.23	<0.01*
Zhongyuan District in Zhengzhou (economic level – H)	4,017	34	0.846 ± 0.28	
Longting District in Kaifeng (economic level – L)	3,907	54	1.382 ± 0.37	0.02**
Rural area	11,792	57	0.483 ± 0.12	
Zhongmu County (economic level – H)	4,180	33	0.789 ± 0 .27	
High-tech zone in Puyang City (county) (economic level – L)	3,894	6	0.154 ± 0.12	<0.01***
Mengjin County (economic level – M)	3,718	18	0.484 ± 0.22	

*Comparison of asthma prevalence between residents in urban and rural areas. **Comparison of prevalence in different urban areas. ***Comparison of asthma prevalence among residents in different rural areas.

Prevalence of asthma among urban residents (Longting District of Kaifeng, Zhongyuan District of Zhengzhou) was 1.1% ± 0.23% (88/7,924), whereas that in rural residents (high-tech zone in Puyang, Mengjin County, and Zhongmu County) was 0.48% ± 0.12% (57/11,792). A significant difference was found for prevalence of asthma between rural and urban areas (*Χ*^2^ = 25.13, *P* < 0.01). The prevalence of asthma among urban residents in areas with different economic development levels was significantly different (*Χ*^2^ = 5.176, *P* = 0.02). The prevalence of asthma among rural residents in areas with different economic development levels was significantly different (*Χ*^2^ = 16.92, *P* < 0.01).

### Prevalence of asthma in different age groups

The survey, which included 2,857 children, showed that 13 children had asthma. The prevalence of asthma in children was 0.49% ± 0.26%. A total of 17,004 participants were adults aged ≥14 years, among which 131 had asthma. The prevalence of asthma in adults was 0.77% ± 0.13%. No significant difference was observed between the prevalence rate of adults and children (*P* = 0.10). Table 5 shows the prevalence of asthma in the different age groups.

**Table 5:** **Asthma prevalence in the different age groups**

Age group (years)	No. of samples	Asthma cases	Prevalence (%)	***P*** value
Children				
0–13	2,694	13	4.8 ± 2.6	
Adults	17,167	131	7.6 ± 1.3	0.10*
14–25	2,982	13	4.3 ± 1.2	
26–45	6,597	30	4.5 ± 2.3	
46–65	5,328	49	9.2 ± 2.5	
66–75	1,541	21	13.6 ± 5.7	
≥76	719	19	26.4 ± 11.7	
Total	19,861	145	7.3 ± 1.2	

*Comparison of prevalence rates between adults and children.

### Medications used for treating asthma

Among 154 patients with asthma, only 33.8% (n = 52) received asthma medication regularly. Among patients who received regular treatment, 25% (n = 13) used oral glucocorticoids, 71.1% (n = 37) used oral theophylline, 9.6% (n = 5) used oral leukotriene modifiers, 3.8% (2) used oral short-acting β_2_ receptor agonist, 5.8% (n = 3) used oral long-acting β_2_ receptor agonist, 17.3% (n = 9) used glucocorticoid inhalation, 7.7% (n = 4) used long-acting β_2_ agonist inhalation, 7.7% (n = 4) used short-acting β_2_ receptor agonist inhalation, 1.9% (n = 1) used intravenous corticosteroids, and 1.9% (n = 1) used intravenous glucocorticoids. Among the 52 people who received regular treatment, 32.7% (n = 17) used a combination of two or more kinds of medicines. The most common combination therapy was oral corticosteroids and oral theophylline (n = 7 cases). Seven cases inhaled corticosteroids, and only two cases used medication combined with inhaled short-acting β_2_ receptor agonist.

### Asthma control

**Figure 2:**
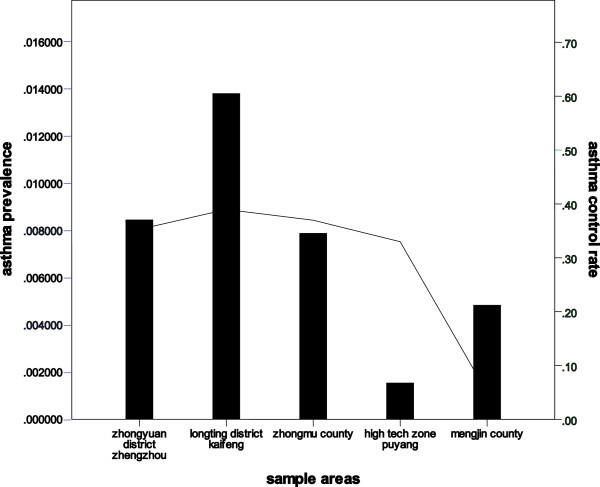
**The bars show asthma prevalence, and the curve shows asthma control rate in the sampling areas.** Asthma control rate was computed as the ratio of controlled to partially controlled and uncontrolled asthma sufferers.

All 145 asthma patients received clinical evaluation of asthma control by symptom-based guidelines. The controlled level was achieved in 48 (33.1%) of the patients. A total of 72 (49.7%) patients achieved partially controlled level, and 25 (17.2%) patients were classified as uncontrolled. Patients receiving regular treatment accounted for 38.5% (*n* = 20) in the controlled group and 61.5% in the partially controlled and uncontrolled groups. Among patients without regular treatment, 27.5% (*n* = 28) had controlled asthma and 72.5% had partially controlled and uncontrolled asthma. Patients who received medicines regularly and those who did not showed significant difference in asthma control.Asthma control rate among urban residents living in Zhongyuan District of Zhengzhou and Longting District of Kaifeng City was 0.35 and 0.39, respectively. Asthma control rates among rural residents living in Zhongmu County, high-tech zone in Puyang, and Mengjin County were 0.37, 0.33, and 0.05, respectively (Figure 2). The level of asthma control in Mengjin County was lower compared with that of the other sample areas.

Table 6 presents the characteristics of asthma sufferers according to the level of control. Among patients with partially controlled and uncontrolled asthma, untreated patients comprised a higher percentage than treated patients did (41.93% vs. 20.77%, p < 0.01). Patients in urban areas showed a higher rate of controlled asthma than those in rural areas (20.78% vs. 10.39%, p < 0.01).

**Table 6:** **Characteristics of asthma sufferers by control classification**

	Medication		Educational status		Age		Area		Smoking history	
	Treated (% asthma)	Untreated (% asthma)	College degree (% asthma)	Without college degree (% asthma)	Child (% asthma)	Adult (% asthma)	Urban (% asthma)	Rural (% asthma)	Never smoked	Smoke or ever smoked
Controlled	20 (13.79%)	28 (18.18%)	1 (0.65%)	47 (30.52%)	2 (1.30%)	11 (7.14%)	32 (20.78%)	16 (10.39%)	34	14
Partially controlled and uncontrolled	32 (20.77%)	65 (41.93%)	8 (5.19%)	89 (57.79%)	46 (29.87%)	86 (55.84%)	56 (36.36%)	41 (26.62%)	44	28
P value	P < 0.01		P = 0.02		P = 0.132		P < 0.01		P = 0.01	

## Discussion

This cross-sectional observational study showed that the prevalence rate of asthma in Henan Province, China was 0.73%, whereas a prevalence rate of 1.05% was reported in 2000 by Wang et al. [9]. The prevalence of asthma in other areas in China varied widely, with only 0.38% in Qinghai Province [14] and 0.94% in Guangdong Province [15]. Compared with previous studies, our research focused more on the differences in asthma prevalence between urban and rural areas and prevalence in areas with different economic levels. We determined the relationship between socioeconomic status and asthma prevalence. As reported previously, asthma comprises a range of heterogeneous phenotypes that differ in presentation, etiology, and pathophysiology. The risk factors for each recognized phenotype of asthma are complex and include genetic, environmental, and host factors. In terms of social environment and lifestyle, the increase in the prevalence of asthma was suggested to be related to modern Western culture [16, 17]. Chinese lifestyle is transitioning from traditional to modern more quickly and at a shorter period than in many other countries [18]. Compared with the data (prevalence rate = 1.05%) obtained 10 years ago, the trend in the prevalence of asthma in Henan Province did not increase. This result may be related to the under-developed economy of Henan province. The province has a rural population of nearly 60% [11], and this population retains the traditional lifestyle. The role of outdoor air pollution in causing asthma remains unclear [3]. Previous studies have shown that the prevalence rate of asthma among city residents was 1.1%, which was significantly higher than that in rural areas (0.48%). Outdoor environmental pollution may be the main reason for this phenomenon. A higher concentration of harmful gas and fine particulate matter (PM2.5) is present in outdoor air in cities than in rural areas. PM2.5 is not only associated with increasing asthma prevalence among children with related emergency and hospitalization [19], but also with adult-onset asthma [20].

In this study, we found that the prevalence of asthma in a city with a high-level economy was lower than that in a city with a low-level economy. By contrast, prevalence of asthma in a rural area with a high-level economy was higher than that in a rural area with a low-level economy. Previous studies have indicated that the prevalence of asthma is positively associated with socioeconomic status, and the incidence rate in families is negatively associated with economic condition [21, 22]. However, other studies showed contradictory conclusions [23, 24]. Given that social economic status comprises numerous factors including geographical environment, air pollution, and health habits, determining the relationship between socioeconomic status and asthma incidence is difficult [25, 26].

We studied the asthma control issues in rural and urban areas, including medication used and level of disease control among asthma sufferers in these areas. We found that patients receiving long-term treatment accounted for only 33.8%, and that the most commonly used medications were oral glucocorticoids and theophylline. Only two in 145 patients were treated by inhaled corticosteroids combined with rapid-acting β2 receptor agonists. These results showed that asthma management was poor. Implementation of general practitioners, specialists’ consultation, and patients’ education according to the guideline has been strengthened to improve the diagnosis and treatment of asthma in Henan province.

In this study, we used symptom-based guidelines to assess the disease control level of asthma patients. The patient classifications according to the symptom-based guidelines were as follows: controlled, 33.1%; partially controlled, 49.7%; and uncontrolled, 17.2%. Partially controlled and uncontrolled rates accounted for 66.9%. In Canada, asthma control was administered to ~53% of adults in the age range of 18 years old to 54 years old who reported having this disease; asthma control was assessed using symptom-based guidelines [27]. In eight European countries and in Canada, Australia, and the USA, the rate of uncontrolled asthma was 51%; asthma control was assessed using the Asthma Control Questionnaire [28]. In France, Germany, Italy, Spain, and the UK, the uncontrolled rate was ~50%, and asthma control was assessed with the Asthma Control Test [29]. In Henan Province, insufficient management of asthma may be among the reasons for poor asthma control.

In this study, asthma patients were divided into two groups according to control level (Table 6) to determine the differences between the controlled group and the uncontrolled and partially controlled groups. By comparing age, education level, smoking history, regular treatment, and living area, we found that a higher proportion of urban residents and a larger number of sufferers receive regular treatment in the controlled group than in the partially controlled and uncontrolled groups. This finding indicates that urban residents may have access to better treatments than rural residents, and the disease control rate may be higher in urban areas than in rural areas.

This study has limitations and potential biases. Data are limited to one province of China, and these data may not represent the situation in the other Chinese provinces that are geologically different from Henan. The prevalence may also be underestimated because the diagnoses of some patients may have been based on self-reported data or the criteria for asthma may have been overestimated, leading to false positives and false negatives. However, the prevalence results obtained in this study were consistent with those obtained in another study [9]. Biases also exist in asthma control assessment because three children aged below five years old were included in this study and the asthma control questionnaires were completed by their parents.

## Conclusion

The study showed that the prevalence of asthma in Henan Province, China was very low. Prevalence of asthma among urban residents was higher than among rural residents. Differences in asthma prevalence were also observed among urban and rural residents living in areas with different economic development levels. Treatment for asthma was insufficient in both urban and rural areas, resulting in suboptimal asthma control results. Future studies should focus on educating primary care physicians and specialists on the importance of asthma control and implementation of the GINA guidelines.

### Consent

Written informed consent was obtained from the patient for the publication of this report and any accompanying images.

## Acknowledgements

This work was supported by People’s Hospital of Henan Province, Zhengzhou University.
